# Effectiveness of tnf inhibitors therapy in children with juvenile idiopathic arthritis aged 2 to 5 years

**DOI:** 10.1186/1546-0096-12-S1-P344

**Published:** 2014-09-17

**Authors:** Violetta Opoka-Winiarska, Jacek Postępski, Agnieszka Korobowicz-Markiewicz, Aleksandra Szabat, Andrzej Emeryk

**Affiliations:** 1 Department of Pediatric Pulmonology and Rheumatology, Medical University of Lublin, Poland, Lublin, Poland

## Introduction

Juvenile idiopathic arthritis (JIA) therapy in the youngest patients (pts) is particularly difficult. Etanercept (ETA) and adalimumab (ADA) are approved for use in moderate and severe JIA in pts older than 2 years, but available data about treatment in the youngest children are limited.

## Objectives

The aim of study was the evaluation of the effectiveness of TNF inhibitors in pts with JIA who had started therapy before the age of five.

## Methods

13 children aged 2 to 5 years with polyarticular JIA - 2 or extended oligoarticular JIA -11 pts (according to the ILAR criteria) treated with anty-TNF were included in the study (2007-20013). All pts at the start of anti-TNF treatment received two synthetic modified drugs (including methotrexate) and glucocorticoids (GC), in 10 pts intraarticular GC injections were used. The assessment of clinical effectiveness included JIA outcome parameters (PhGA, PaGA, CHAQ, ESR/CRP, number of joints with active arthritis; number of joints with limited range of motion), JADAS 71 scale and American College of Rheumatology pediatric (PedACR) 30/50/70/90 responses. Disease activity was measured at the baseline and every 6 months.

## Results

In all (13) pts the first used biological drug was ETA. The treatment of ETA was started in a mean 2,46 year ± 1,2 months of disease. The mean duration of therapy with ETA was 24 ± 11,81 months. At 6 months of ETA treatment 85% of pts achieved PedACR30, high PedACR 50/70/90 responses were achieved in 61% pts. In 8 pts ETA treatment was terminated after at least 18 months remission on the drug (according to the Polish therapeutic program). 4 of them developed a disease exacerbation in mean 3,25 months after the termination of ETA and the treatment was restarted. 2 children (15%) did not respond satisfactorily and they were switched to ADA and at least PedACR 30 response was achieved. In 11 children the treatment of GC was terminated. In all patients the treatment with anti-TNF was well tolerated. No deaths, malignancies or opportunistic infections were reported.

## Conclusion

In the youngest children with JIA, TNF inhibitor therapy is well tolerated and effective. In case of active disease it is necessary to switch to another biological agent.

## Disclosure of interest

None declared

